# Effects of Pore Size and Crosslinking Methods on the Immobilization of Myoglobin in SBA-15

**DOI:** 10.3389/fbioe.2021.827552

**Published:** 2022-01-28

**Authors:** Hengmin Miao, Maosheng Li, Xiaochun Sun, Jikun Xia, Yanqing Li, Jiao Li, Fang Wang, Jiakun Xu

**Affiliations:** ^1^ School of Materials Science and Engineering, Shandong University of Technology, Zibo, China; ^2^ Key Lab of Sustainable Development of Polar Fisheries, Lab for Marine Drugs and Byproducts of Pilot National Lab for Marine Science and Technology, Yellow Sea Fisheries Research Institute, Chinese Academy of Fishery Sciences, Qingdao, China; ^3^ School of Food Science and Engineering, Qilu University of Technology (Shandong Academy of Sciences), Jinan, China; ^4^ Marine Science Research Institute of Shandong Province, Qingdao, China; ^5^ College of Marine Science and Biological Engineering, Qingdao University of Science andTechnology, Qingdao, China; ^6^ College of Food Science and Technology, Shanghai Ocean University, Shanghai, China

**Keywords:** myoglobin, SBA-15, immobilization, catalytic activity, reusability

## Abstract

A series of stable mesoporous silica sieves (SBA-15) with different pore sizes (9.8, 7.2, and 5.5 nm) were synthesized using a hydrothermal method. The resulting mesoporous material was then utilized for protein immobilization using myoglobin (Mb) as the target protein. The effects of pore size and adsorption methods on the immobilization efficiency of Mb in a mesoporous material were studied. The SBA-15 with a pore size of 7.2 nm showed the best loading capacity, reaching 413.8 mg/g. The SBA-15 with a pore size of 9.8 nm showed the highest retained catalytic ability (92.36%). The immobilized enzyme was more stable than the free enzyme. After seven consecutive assay cycles, Mb adsorbed by SBA-15 (Mb/SBA-15) and Mb adsorbed by SBA-15 and crosslinked with glutaraldehyde (Mb/G/SBA-15) retained 36.41% and 62.37% of their initial activity, respectively.

## Introduction

Conventional enzymes are especially effective when catalyzing a series of reactions under mild conditions. However, under cruel conditions, conventional enzymes have obvious limitations in practical applications due to insufficient stability (Schmid et al., 2001; Sheldon, 2007; Wang and Hsieh, 2008; Badgujar and Bhanage, 2015). Most enzymes are unstable and lose their activity easily in organic solvents or high-temperature conditions, and they are difficult to be recycled, so they are not always ideal for industrial applications. These disadvantages would be overcome by immobilizing enzymes on solid supports (Hartmann, 2005; Clark et al., 2006; Vinu et al., 2006; Cipolatti et al., 2014; Eş et al., 2015). Over the past few decades, great efforts were devoted to constructing immobilized protein for improved efficiency and stability (Bezbradica et al., 2006; Zheng et al., 2006). To date, several new types of carriers and technologies on immobilization have been developed to enhance the loading and stability of enzymes, which could reduce the cost of enzyme biocatalysts for industrial applications. These include crosslinked enzyme aggregates, microwave-assisted immobilization, mesoporous supports, and click chemistry technology (Xu et al., 2014). Mesoporous silica materials have gathered significant attention in both academic and industrial areas due to their well-ordered and adjustable pore structures as well as abundant hydroxyl groups, accompanied by a series of advantages, such as cost-effectiveness, simple process, readily available raw materials, easy purification of products, low cost, and long guarantee period (Salis et al., 2005). There are mainly three kinds of immobilization methods: physical adsorption, where van der Waals forces, hydrogen bonding, and electrostatic force exist between the support and enzyme; crosslinking adsorption, where the active group of the enzyme reacts with a crosslinking agent to form crosslinked aggregates; and covalent adsorption, where chemical bonds are formed between support and enzyme. Physical adsorption is one of the most widely used methods due to its simplicity, and it typically does not change the native structure of the enzyme (Li et al., 2009; Jesionowski et al., 2014).

SBA-15 is a form of mesoporous silica nanoparticle with uniform pore size and long-range ordered pore structure. The morphology, pore size, and pore structure of mesoporous silica can be freely designed and controlled. SBA-15 has been extensively investigated as a catalyst supporting absorbents and drug delivery due to its simple preparation, uniform pore structures, diverse morphology, and well-modified surface properties. On the one hand, the large surface area and high pore volume of SBA-15 not only can promote the immobilization of enzyme molecules but also can protect the enzyme and enhance its operational stability (Lynch et al., 2016). On the other hand, in the process of adsorption, too large a pore may make the proteins easier to leach out from the channels, resulting in a decrease in enzyme loading (Kang et al., 2007). According to reports, the ratio of pore size to the size of the enzyme may greatly affect the adsorption of the enzyme (Lü et al., 2008), mainly because the smaller pore size makes it difficult for proteins to enter the channel, resulting in a low enzyme loading. However, in some other cases, the nature of the pores, total pore volume, and surface area have no obvious effects on the enzyme immobilization process (Serra et al., 2008).

Although the physical adsorption method is simple, the stability of the immobilized enzyme might be poor due to the leaching of adsorbed enzymes. For instance, Serra et al. studied the loss of physical adsorption lipase and found that nearly 30% of the enzyme was leached from the support silica. Hydroxyl groups are the only existing groups on the surface of silica; the silica support generally requires further modification with functional groups (like –NH_2_ or –CHO groups) to generate chemical bonds for enzyme immobilization, but it required harsh conditions and multistep synthetic processes (Hong et al., 2008). Crosslinking adsorption and covalent adsorption are common methods to overcome this drawback. Compared with covalent adsorption, crosslinking adsorption is simpler and easier to operate, and this method depends on the use of coupling reagents (such as polyamines, polyethyleneimine, and various phosphates) to crosslink the enzyme molecules. Chemical means of crosslinking normally involve covalent bonding between enzymes by using reagents such as glutaraldehyde or toluene diisocyanate. Glutaraldehyde is a common crosslinking agent, and its aldehyde group could react with the amino groups in enzyme molecules to realize the crosslinking between immobilization enzymes. Kim et al. (2010) adsorbed the enzyme onto mesoporous silica and then add glutaraldehyde to crosslink the enzymes with the improved recovery of immobilized enzyme. Gao et al. (2010) used glutaraldehyde as a crosslinking agent to adsorb lipase on SBA-15; the immobilized lipase retained 80.5% activity after repeated use for 6 times.

Myoglobin (Mb), having molecular dimensions of 4.5 × 3.5 × 2.5 nm (Wüstneck et al., 1989), is a heme protein that originally functions as oxygen storage and transportation. Mb can be transformed into peroxidase by protein engineering, which can catalyze various reactions such as oxidation of guaiacol and indole (Xu et al., 2012; Liao et al., 2020) and decolorization of industrial dyes (Zhang et al., 2019). In this study, three different pore diameters of SBA-15 (5.5, 7.2, and 9.8 nm) were prepared using a reported method (Zhao et al., 1998a; Zhao et al., 1998b) and characterized by means of scanning electron microscopy (SEM), Fourier transform IR (FTIR) spectroscopy, small-angle X-ray diffraction (SAXRD), and nitrogen adsorption–desorption experiments. The synthesized SBA-15 was then used for Mb immobilization. The influences of pore size and surface area of SBA-15 on the protein immobilization were investigated. Glutaraldehyde as a crosslinking agent has been utilized in the immobilization process, which effectively prevented the loss of enzyme molecules from the pore channels of mesoporous materials.

## Materials and Methods

### Materials

1,3,5-Trimethylbenzene (TMB) and P123 triblock copolymer (poly(ethylene oxide)-block-poly(propylene oxide)-block-poly(ethylene oxide), EO20-PO70-EO20, Mav = 5,800), and tetraethyl orthosilicate (TEOS) were purchased from HI-LEAD BIO-TECHNOLOGY, Qingdao, China. Guaiacol was purchased from TCI, Shanghai, China. H64D/V68I Mb was expressed in *Escherichia coli* BL21 (DE3) cells and purified using the procedure described previously.

### Methods

#### Synthesis of SBA-15

The SBA-15 mesoporous materials were synthesized using a previously reported method (Zhao et al., 1998a; Zhao et al., 1998b), with P123 as a structure-directing agent and the TMB as a pore dilator agent. First, 4 g of P123 was dissolved in 104 ml of water and 20 ml of 2 M HCl by stirring at 35°C. Then, an appropriate amount of TMB was added to adjust the final target pore size. A total of 8.5 g of TEOS was then added to the solution and stirred at 35°C for 24 h. This resulting solution was transferred into a Teflon-lined autoclave and heated to 100°C for 24 h. Finally, the solid products were filtered and washed with deionized water, air-dried at room temperature, calcined at 500°C for 6 h at a heating rate of 1°C min^−1^ in a tube furnace, and returned to room temperature at a cooling rate of 5°C min^−1^.

#### Characterization of SBA-15

SEM micrographs were obtained on a Zeiss Merlin compact field-emission SEM (Carl Zeiss, Oberkochen, Germany). Nitrogen adsorption–desorption experiments were performed using an ASAP 2460 Micromeritics System, Micromeritics instrument Ltd, America. Before the measurement, samples were degassed in a vacuum at 200°C for 2 h. The surface areas were calculated using the Brunauer–Emmett–Teller (BET) method based on the desorption isotherms, and the pore diameter distributions were calculated from desorption branches using the Barrett–Joyner–Halenda (BJH) method. The small-angle X-ray powder diffraction patterns were obtained using Cu Kα radiation on a Bruker D8 Advance diffractometer (Bruker, Kontich, Belgium). The data were collected from 0.5° to 10° with a step size of 0.5°. The IR spectra were recorded in diffuse reflectance mode (spectral resolution 4 cm^−1^, 100 scans) using FTIR spectroscopy (Shimadzu 8201 PC spectrophotometer in the region 400–4,000 cm^−1^; Shimadzu Corp., Kyoto, Japan). The samples were prepared using the standard KBr disk method. All analyses were conducted under dry conditions.

#### Adsorption of Myoglobin on SBA-15

The optimal conditions for adsorption were obtained by changing the immobilization time, shaking speed, temperature, and pH. The adsorption experiments were performed by mixing 5 mg of SBA-15 and 1 ml of protein solution (2 mg/ml) and stirring at 25°C for 15 h in 2 ml of phosphate buffer (50 mM, pH 6). Then, the reaction mixture was centrifuged, the supernatant was removed, and 1 ml of phosphate buffer (50 mM, pH 6) was added to the precipitate. The mixture was stirred for 1 h to wash the SBA-15 and centrifuged, and the supernatant was removed. This last wash step was repeated three times. The UV absorption value of the supernatant was measured at 470 nm to determine the concentration of protein in the supernatant. The final products were identified as Mb/SBA-15. The kinetics of the adsorption process was investigated by testing the relationship between the loading amount of Mb enzyme originally added and time.

To determine the crosslinking adsorption of Mb onto SBA-15, 2.0 wt% of glutaraldehyde was added to the solution after 15 h of Mb protein physical adsorption, and the mixture was stirred for 30 min at 25°C to crosslink the adsorbed protein. The final product was identified as Mb/G/SBA-15. To determine the effect of glutaraldehyde dosage on the activity of the immobilized enzyme, different volumes of glutaraldehyde were used in the protocol described for enzyme crosslinking, and the activity was compared with that of Mb/SBA-15. To determine the effect of pH on protein adsorption, 0.5 mg of SBA-15 and 1 ml of protein solution were added to 2 ml of buffer (pH 3–5, 50 mM, citrate buffer; pH 6–9, 50 mM, phosphate buffer) following the procedure described above.

#### Catalytic Activity of Free Myoglobin and Immobilized Myoglobin

The assays comparing the catalytic activity of free Mb and immobilized Mb were performed using the oxidation of guaiacol and the product by UV absorption at 470 nm (Zhang et al., 2020). The reaction mixture (50 mM of phosphate buffer, 1 μM of Mb, 2.5 mM of guaiacol) was incubated at 25°C for 15 min, and the reaction was initiated by the addition of H_2_O_2_ (final concentration 10 mM). The effect of pore size on the activity of the immobilized enzyme was determined by the retained activity, which is the percentage of activity that is maintained in the immobilized enzyme compared with the free enzyme activity.

#### Operational Stability Test for Free and Immobilized Myoglobin

To assess the stability of the immobilized protein, the influence of metal ions (Ba^2+^, Ca^2+^, Mg^2+^, Mn^2+^, and Al^3+^), organic solvents (methanol, ethanol, isopropanol, glycol, and formic acid), storage time, and temperature on Mb activity were studied. The reactions were carried out in 50 mM, pH 6, of phosphate buffer.

The experiment to study the effect of metal ions on Mb activity was performed as follows: free Mb, Mb/SBA-15, and Mb/G/SBA-15 were incubated for 1 h in a buffer solution (phosphate buffer, 50 mM, pH 6) containing different metal ions (10 mM), with a control group containing solely buffer solution. Then, the enzyme activity was tested using the method described in *Catalytic Activity of Free Myoglobin and Immobilized Myoglobin*. The effect of organic solvents (10% v/v) was investigated in the same way. The effect of temperature on the activity of free and immobilized Mb was examined by incubation for 1 h in phosphate buffer (50 mM, pH 6) in a temperature range between 10°C and 60°C. The effect of storage time was tested by determining the residual activity after storing the Mb in phosphate buffer (50 mM, pH 6) for different time periods of 30 days at 25°C. The activity was assayed every 5 days.

#### Reusability Test for Myoglobin/SBA-15 and Myoglobin/G/SBA-15

The proteins were physically adsorbed onto the SBA-15 with different pore sizes, and then the catalytic activities were tested by measuring the oxidation of guaiacol. After centrifugation of the reaction solution, the supernatant was tested using UV, and the precipitate was washed three times with phosphate buffer (50 mM, pH 6). The above method was used to test the reusability of Mb/SBA-15 for seven consecutive assays. The remaining activity was calculated by comparing it with the first assay. The leaching amount of protein in the supernatant of the assays for Mb/SBA-15 and Mb/G/SBA-15 was determined to calculate the protein leaching rate (leaching amount of protein/initial total amount of immobilized protein) after each reaction.

## Results and Discussion

### Structural Characteristics of the SBA-15

**FIGURE 1:**
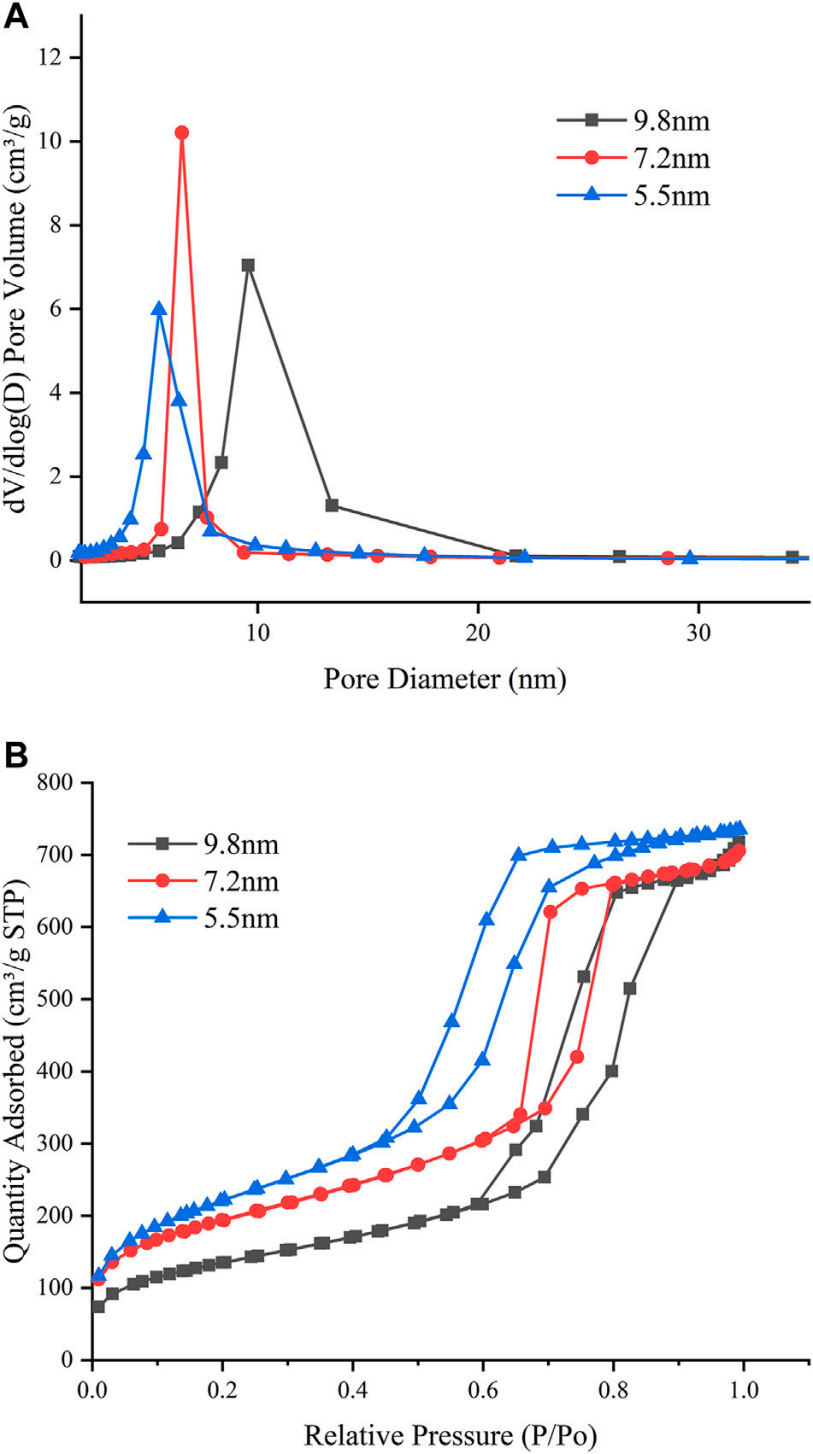
**(A)** BJH pore-size distributions. **(B)** Nitrogen adsorption-desorption isotherms.

The BJH pore-size distributions and nitrogen adsorption–desorption isotherms of materials are shown in Figure 1. The BJH pore-size distributions show that the pore sizes of the three samples are 9.8, 7.2, and 5.5 nm. The nitrogen adsorption–desorption isotherms of the three samples can be classified as type IV and exhibit an H1-type hysteresis loop at high relative pressure, which is a typical feature of hexagonal cylindrical pore mesoporous materials (Shah et al., 2008). Detailed structural features are shown in Table 1.

**TABLE 1:** Detailed structural features of SBA-15.

BJH pore diameter (nm)	BJH pore volume (cm^3^/g)	BET surface area (m^2^/g)
9.8	1.08	484.133
7.2	1.05	612.911
5.5	1.02	803.489

Note. BJH, Barrett–Joyner–Halenda; BET, Brunauer–Emmett–Teller.

SAXRD patterns of the SBA-15 show three well-resolved peaks (Supplementary Figure S1) that are indexed as (100), (110), and (200) reflections. These three peaks are the characteristic of hexagonal mesoporous structures, are associated with p6mm hexagonal symmetry (Zhao et al., 1998a), and indicate that the structure of the SBA-15 is actually representative of a long-range order.

The SEM images of SBA-15 are shown in Supplementary Figure S2, showing that the structures of the three samples are the same. The structure of materials is rod-shaped; meanwhile, they consist of many rod-shaped domains with relatively uniform sizes.

### Immobilization of Myoglobin on the SBA-15 Using Physical Adsorption

We assessed the effect of SBA-15 pore diameter (5.5, 7.2, and 9.8 nm) on the immobilization efficiency and activity of Mb compared with free Mb using a physical absorption method. The influence of pore size on Mb loadings on the SBA-15 is shown in Supplementary Figure S3. As shown in Supplementary Figure S3A, the SBA-15 with a larger pore diameter showed much higher adsorption efficiency, indicating that the material with larger pores is more efficient for protein adsorption, whereas the smaller pore size is not conducive to the entry of protein molecules into its channels and thus limits the spread of the enzymes (Bonzom et al., 2018). Apparently, it takes slightly more time for the adsorbed Mb to move into the channels of the SBA-15. The SBA-15 with a pore diameter of 7.2 nm had better protein adsorption capacity than the other two, with the loading amount reaching 413.9 mg/g, whereas the adsorption capacity of the SBA-15 with pore diameters of 5.5 and 9.8 nm was 381.8 and 359.6 mg/g, respectively (Table 2, initial Mb concentration: 3 mg/ml). This result suggests that the protein immobilized on the material with a larger pore size (9.8 nm) might easily leak out from the SBA-15 during the immobilization process. Supplementary Figure S3B shows that the amount of protein loaded increased with the increase in protein concentration, and the adsorption equilibrium was reached when the protein concentration was more than 2 mg/ml. Although the SBA-15 with a pore size of 7.2 nm was best for adsorption of Mb, the Mb immobilized on the SBA-15 of pore diameter 9.8 nm showed the best remaining enzymatic activity (93.24%). Consistent with the report by Serra (Serra et al., 2008), we also found that the surface area of the SBA-15 had no significant effect on the adsorption capacity.

**TABLE 2:** The maximum adsorption amount and retained activity of Mb onto SBA-15.

BJH pore diameter (nm)	BET surface area (m^2^/g)	Adsorption proteins (mg/g)	Retained activity (%)
9.8	484.133	359.6 ± 10.73	93.24 ± 1.36
7.2	612.911	413.8 ± 9.86	90.10 ± 1.57
5.5	803.489	381.8 ± 9.4	86.13 ± 1.62

Note. BJH, Barrett–Joyner–Halenda; BET, Brunauer–Emmett–Teller.

**FIGURE 2:**
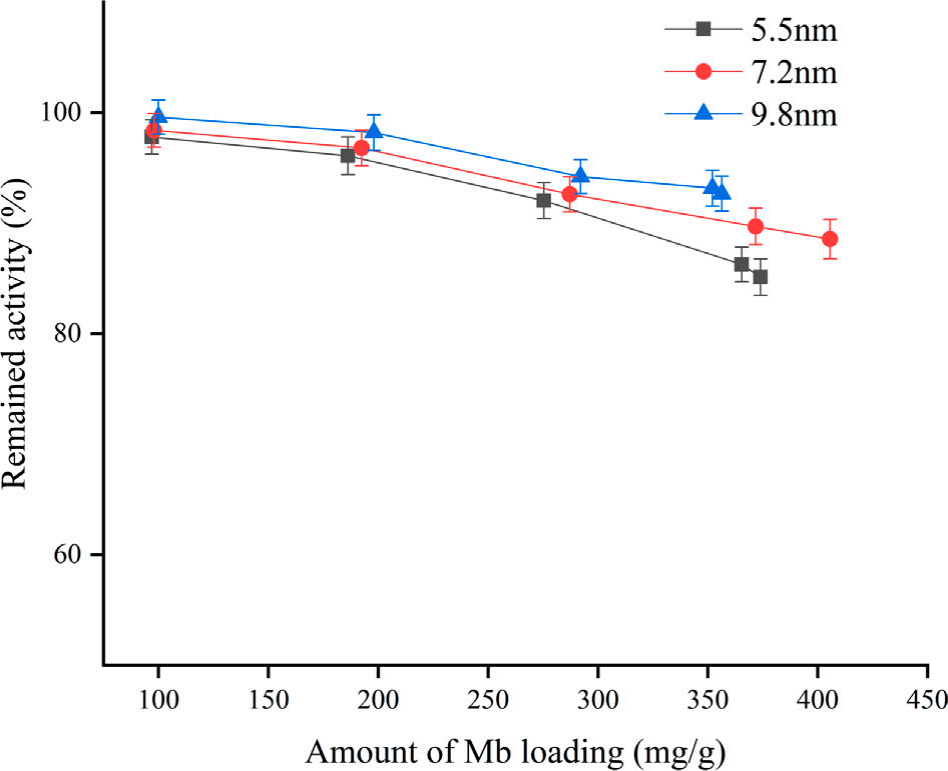
Effect of pore size and amount of Mb loading on remained activity.

As shown in Figure 2, the retained activity of the immobilized protein was affected by the pore size, and the SBA-15 with a larger pore size was best for the enzymatic reaction. On the other hand, the enzymatic activity of immobilized Mb decreased as the amount of Mb loading increased. It is likely that as more and more Mb diffused into the pores of the SBA-15, some might get aggregated and did not distribute as a one layer at the inner surface of the channels, leading to a decrease in enzyme activity per gram due to the non-contact with substrates of the bottom layer Mb (Gao et al., 2010). Considering the effect of pore size on loading capacity and retained activity, the SBA-15 with a pore size of 9.8 nm was the most suitable for enzyme immobilization. Therefore, it was chosen as the representative support for the rest of the study.

### Crosslinking Adsorption of Myoglobin Onto SBA-15

To solve the leaching problem of the adsorbed enzyme, the SBA-15 with a pore size of 9.8 nm was used for crosslinking. Glutaraldehyde was used as a crosslinking agent for adsorption. The addition of glutaraldehyde could reduce the leaching of the enzyme and make the adsorption capacity of the SBA-15 with the 9.8-nm pore diameter reach 471.3 mg/g (Mb/G/SBA-15), which was 31.06% higher than physical adsorption. This is because the aldehyde group of the glutaraldehyde molecule reacts with the amino group of Mb through a Schiff’s base reaction (Chui and Wan, 1997) so that multiple protein molecules are crosslinked to form a whole, which makes it difficult for the protein to leach from the pore. The amount of glutaraldehyde in the absorption process was optimized (Supplementary Figure S4). When the amount of glutaraldehyde was less than 400 μl, the enzyme activity after crosslinking adsorption was the same as that after physical adsorption. However, the enzyme activity was inhibited when the amount of glutaraldehyde solution was more than 400 μl. Therefore, we chose 400 μl of glutaraldehyde solution (2.0 wt%) for the crosslinking adsorption of Mb onto the SBA-15. Too much crosslinking agent could result in a distortion of the enzyme structure (Chui and Wan, 1997), which would lead to the decrease of the catalytic activity.

### Fourier Transform IR Spectrum

**FIGURE 3:**
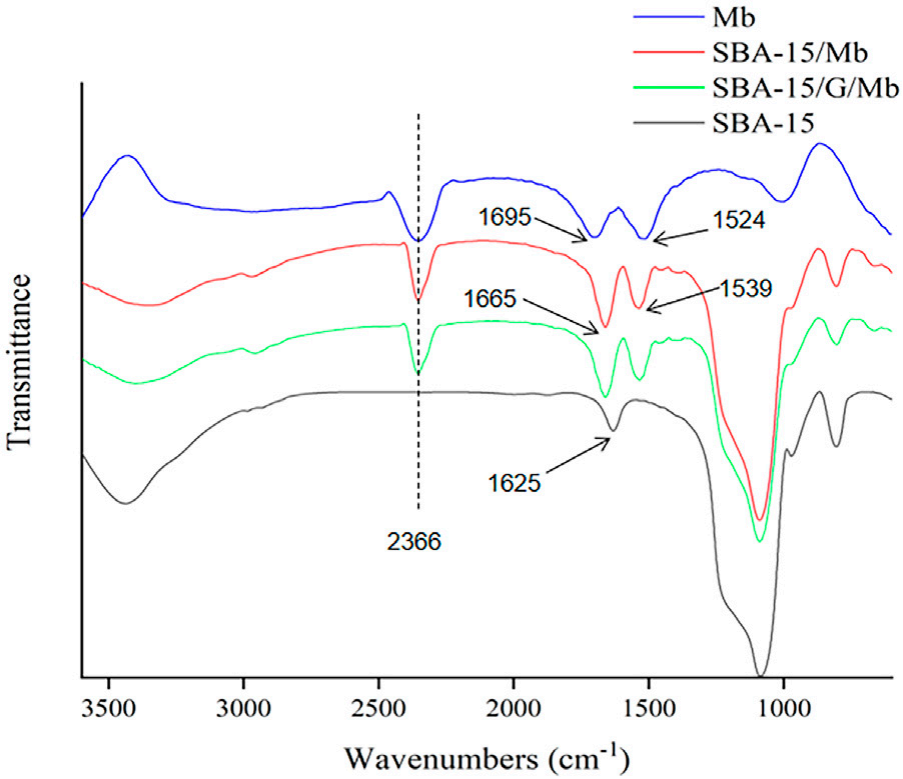
FTIR spectrum.

To confirm that Mb was immobilized on the SBA-15, the FTIR spectra of Mb, SBA-15, SBA-15/Mb, and SBA-15/G/Mb were studied. As shown in Figure 3, in the FTIR spectra of Mb, the peak at 2,366 cm^−1^ corresponds to the secondary amine salt, and the peaks at 1,695 and 1,524 cm^−1^ correspond to amide I and N–H bending vibrations, respectively (Nguyen et al., 2008; Kijima et al., 2018). Compared with that of SBA-15, the absorption of SBA-15/Mb has the characteristic peaks of Mb. The absorption band at about 1,625 cm^−1^ can be attributed to the stretching vibration of Si–OH at the surface of the SBA-15 (Li et al., 2009) because the absorption band of amide I is also near this position. Thus, the two absorption bands overlap, and only one absorption band is evident. FTIR shows that the protein was immobilized to SBA-15/Mb. Meanwhile, the protein was also immobilized on SBA-15/G/Mb, but the characteristic absorption peak of glutaraldehyde was not observed. This may be because the aldehyde group reacts with the amino group in Mb, which also proves the feasibility of glutaraldehyde crosslinking.

### Stability of Free and Immobilized Myoglobin

Supplementary Figure S5 shows the stability of free Mb, Mb/SBA-15, and Mb/G/SBA-15 tested under different conditions. Supplementary Figure S5A shows the thermal stability of both the free and immobilized Mb. The optimum reaction temperature of free Mb was 25°C, whereas both Mb/SBA-15 and Mb/G/SBA-15 showed maximum activities at a higher temperature of 30°C. At 60°C, Mb/SBA-15 and Mb/G/SBA-15 retained 52.98% and 57.5% of the relative activity, respectively, whereas free Mb retained only 31.02%, which proved that the immobilized Mb has higher temperature stability.

The effect of storage time on the relative activity of the free and immobilized Mb is shown in Supplementary Figure S5B. Although the enzyme activity decreased with the increase of storage time, Mb/SBA-15 and Mb/G/SBA-15 retained over 51.5% and 58.9% of the relative activity, respectively, after 30 days, whereas free Mb retained only 33.68%. Thus, the immobilized Mb exhibited better storage stability than free Mb.

Supplementary Figures S5C,D show the effects of metal ions and organic solvents on the activity of free and immobilized Mb, respectively. Considering the effect of metal ions on the enzyme activity, we found that Ba^2+^, Ca^2+^, Mg^2+^, and Mn^2+^ could improve the enzyme activity. Mb/SBA-15 and Mb/G/SBA-15 still retained 19.97% and 21.48% of relative activity, respectively, in the presence of 10 mM of Al^3+^, whereas free Mb was completely inactivated under the same condition. As for the influence of organic solvents on the activity of free and immobilized Mb, the methanol solution could improve the enzyme activity. Ethanol, isopropanol, glycol, and formic acid solutions could inhibit the activity of free and immobilized Mb. The relative activities of Mb/SBA-15 and Mb/G/SBA-15 were higher than those of free Mb under the same conditions. The comparison of the immobilized Mb and free Mb revealed that the relative activity of Mb/SBA-15 was increased by 8.73%, 9.86%, 9.16%, and 30.84% in ethanol, isopropanol, glycol, and formic acid solutions, respectively. Mb/G/SBA-15 retained higher relative activity and increased by 9.2%, 11.64%, 10.28%, and 32.7% in the above solutions. It has been previously observed that Mb immobilized on SBA-15 is protected from metal ions and organic solutions, and the addition of crosslinking agent can further increase the stability of the enzyme.

### Reusability of Myoglobin/SBA-15 and Myoglobin/G/SBA-15

**FIGURE 4:**
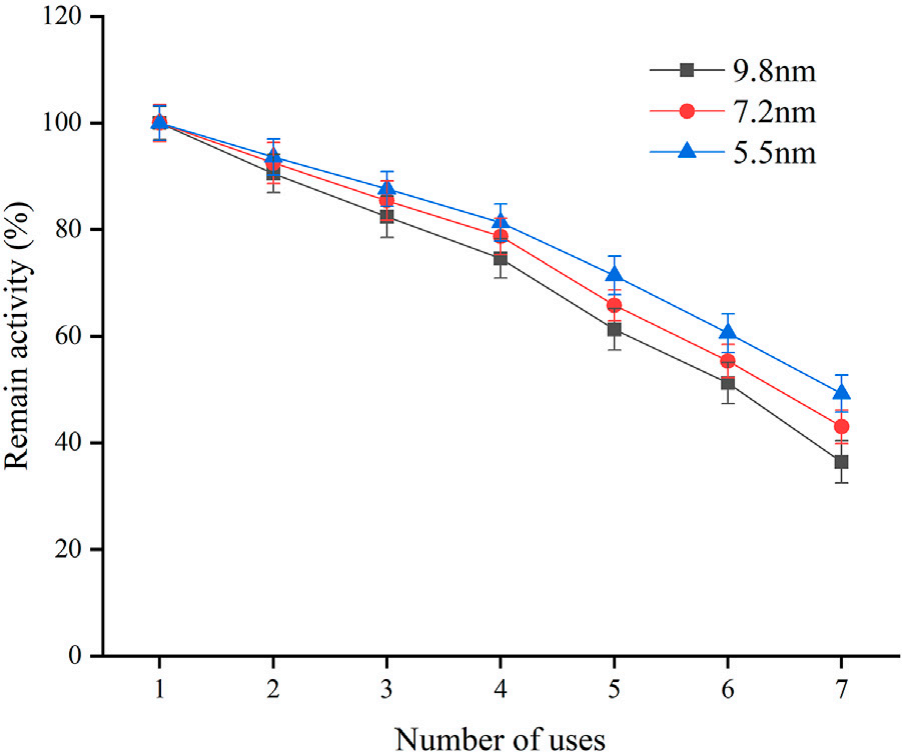
Reusability of immobilized Mb on SBA-15 with different pore diameter.

The reusability of immobilized enzymes is very important for their practical application. Figure 4 shows the remaining activity of Mb immobilized on SBA-15 with different pore diameters after seven consecutive uses. The Mb immobilized on SBA-15 with different pore sizes of 5.5, 7.2, and 9.8 nm retained 49.26%, 43.02%, and 36.41% of its original activities after seven consecutive uses, respectively. The SBA-15 with a smaller pore diameter retained the most activity, which could be attributed to the easy leaking out of the enzyme from SBA-15 with a large pore diameter during the reuse process (Dettori et al., 2018).

**FIGURE 5:**
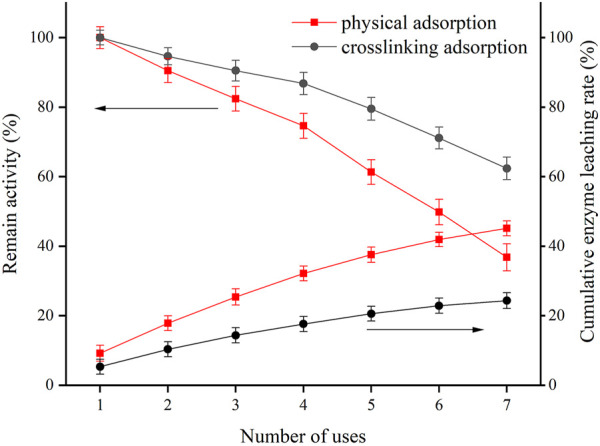
Reusability of immobilized Mb with different methods and immobilized Mb leaching ration.

As shown in Figure 5, the crosslinked adsorption proteins retain more activity, effectively improving enzyme reusability. A total of 62.37% of initial activity was retained after seven consecutive assays, which proved that the addition of glutaraldehyde can reduce the leaching of the enzyme and improve the reusability of the enzyme. As shown in Table 3, the catalytic activity loss of physical adsorption and crosslinking adsorption was 63.59% and 37.63%, respectively. Assessment of the protein content in the supernatant of the eluate revealed that some Mb was leached from the immobilized Mb during the reuse process. The protein leaching rate for Mb/SBA-15 and Mb/G/SBA-15 was 45.12% and 24.31%, respectively. The catalytic activity loss rate was greater than the corresponding protein leaching rate. Therefore, the loss of catalytic activity of immobilized Mb in reuse was caused by protein leaching and partial inactivation of the protein. Thus, crosslinking is a useful method for protein immobilization, which could not only increase the loading ability but also increase the stability of the immobilized protein.

**TABLE 3:** The enzyme activity loss rate and leaching rate after repeated use for seven times with different adsorption methods.

Adsorption method	Enzyme activity loss rate (%)	Enzyme leaching rate (%)
Physical adsorption	63.59 ± 1.46	45.12 ± 1.34
Crosslinking adsorption	37.63 ± 1.38	24.31 ± 1.27

As shown in Supplementary Figure S6, the changes in pH have a large influence on the adsorption capacity by the method of physical adsorption. The reason is that electrostatic force is included in the adsorption process, and the isoelectric points of SBA-15 and Mb are 3.8 and 7, respectively (Essa et al., 2007; Jin et al., 2020). In the zone pI (SBA-15) and pI (Mb), the charges are complementary, and there is an electrostatic attraction between protein and carrier materials, which is conducive to the adsorption of protein on the carrier. As the pH increased from 7 to 9, the overall charge of the protein and SBA-15 is the same, so the protein easily leaches due to electrostatic repulsion. After crosslinking, the effect of pH on protein adsorption decreases, which proves that protein leaching could be reduced by crosslinking.

## Conclusion

We successfully synthesized SBA-15 with different pore diameters using a hydrothermal method. The pore size of the SBA-15 showed significant effects on the immobilization of Mb: a large pore size was conducive to the rapid adsorption of Mb with a better-retained activity. Among the SBA-15 materials used, the SBA-15 with a pore diameter of 9.8 nm was more suitable for the immobilization of Mb. The immobilized Mb showed improved stability against metal ions, organic solutions, and temperature than the free enzyme. The addition of glutaraldehyde for crosslinking during the absorption process increased the adsorption capacity of SBA-15, improving the stability and reusability of immobilized protein. The immobilized Mb, especially the crosslinked version, showed good reuse performance and stability, which is of great significance to the industrial application of Mb.

## Data Availability

The original contributions presented in the study are included in the article/Supplementary Material. Further inquiries can be directed to the corresponding authors.
