# Supplementary material for: Effects of Pore Size and Crosslinking Methods on the Immobilization of Myoglobin in SBA-15
Source: Front Bioeng Biotechnol. 2022 Jan 28;9:827552. doi: 10.3389/fbioe.2021.827552 (PMC8831746; doi:10.3389/fbioe.2021.827552)
Supplement: Supplementary file 1 [file DataSheet1.docx]

Supplementary Material

#
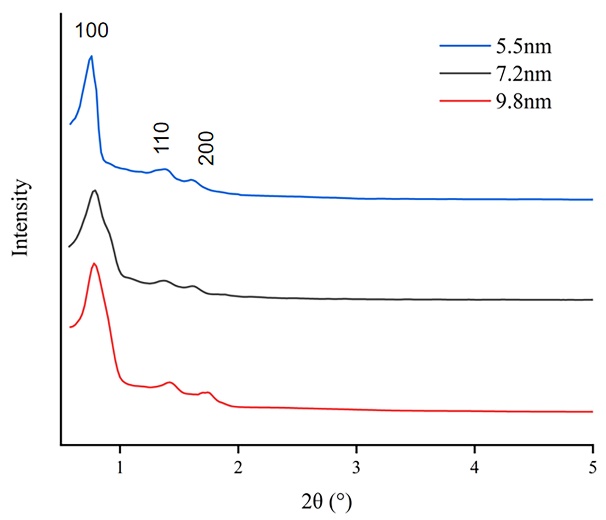
Supplementary Figures

**Supplementary Figure 1.** Small-angle XRD patterns


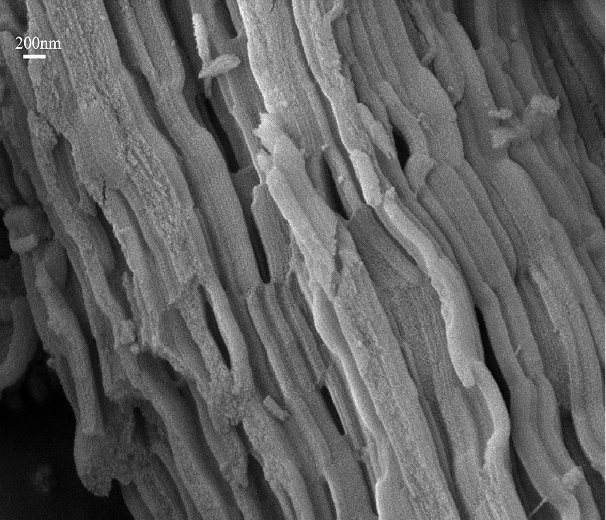

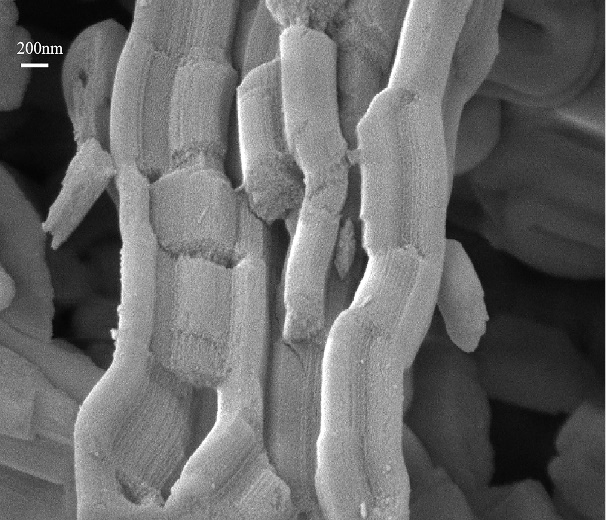

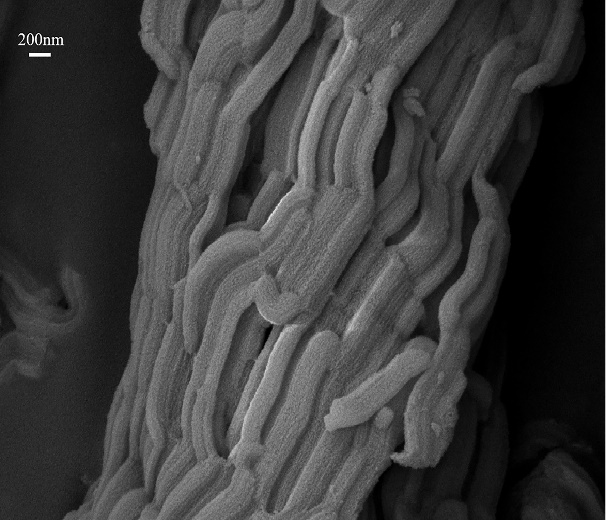
(a) (b)

(c)

**Supplementary Figure 2**. SEM images of SBA-15 (a)5.5 nm; (b)7.2 nm; (c)9.8 nm.


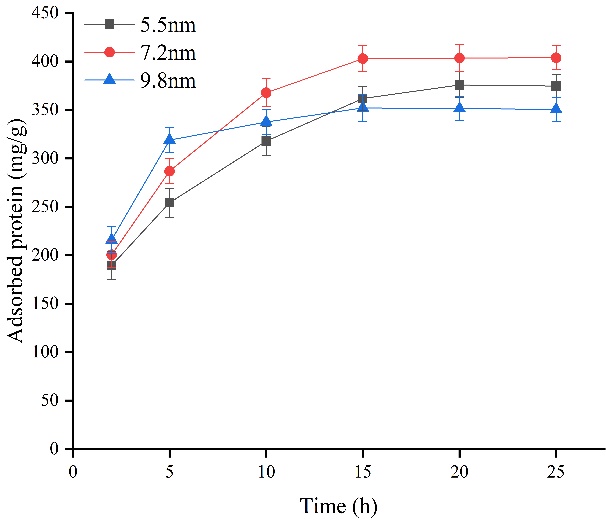

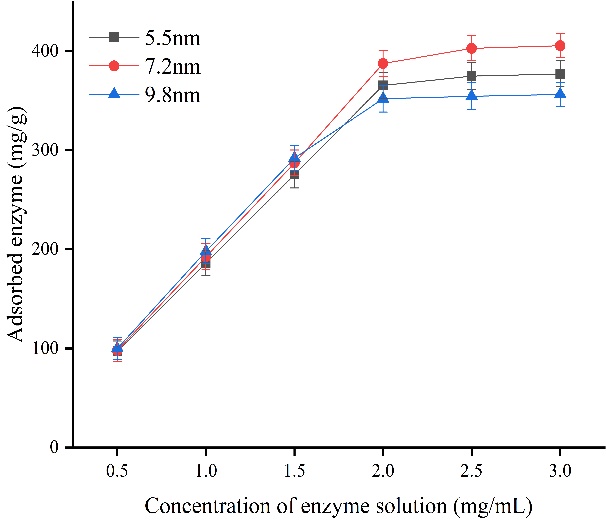
(a) (b)

**Supplementary Figure 3.** Effect of pore size on (a) adsorption kinetics of Mb in SBA-15; (b) Mb adsorption at different enzymes concentrations.

**
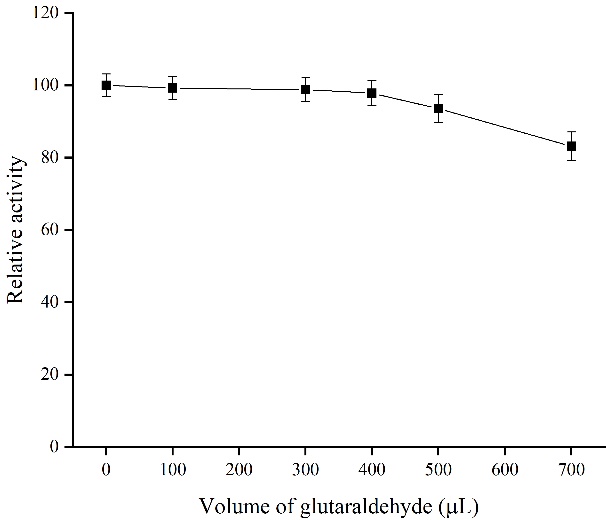
**

**Supplementary Figure 4.** Effect of glutaraldehyde addition on enzyme activity.


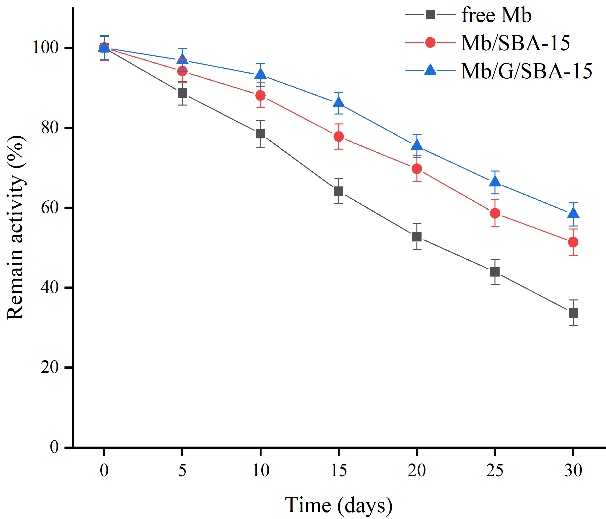

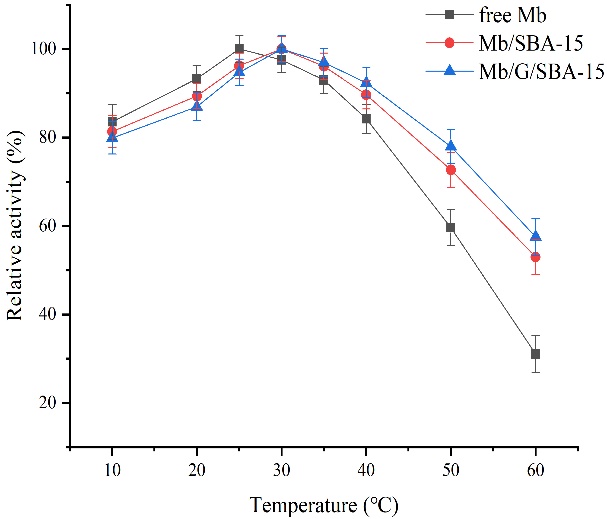


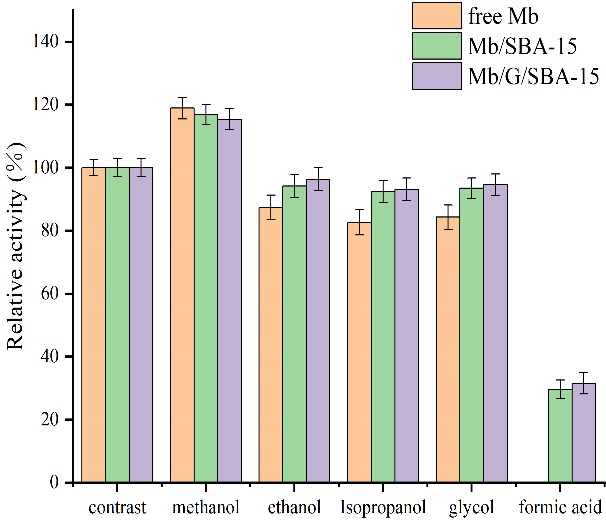
(a) (b)


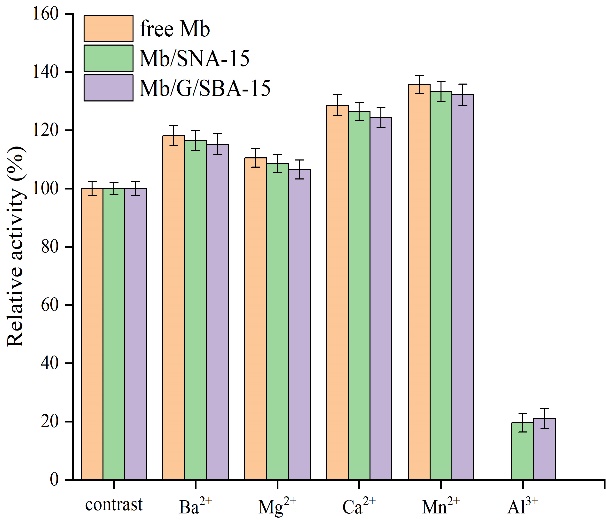
 (c) (d)

**Supplementary Figure 5.** Effect of (a) temperature; (b)storage time; (c) metal ion; (d)organic solutions on the activity of free and immobilized myoglobin.


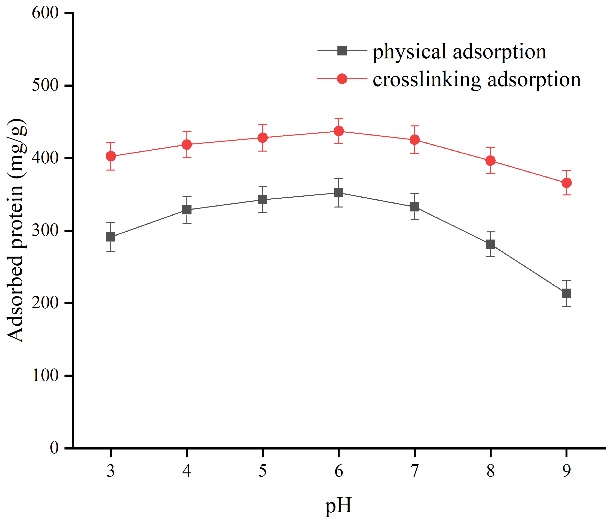


**Supplementary Figure 6.** Effect of pH on the adsorption process of Mb onto SBA-15.
